# Universal Lip Integrity, Proportion, and Structure: A Framework for Achieving Consistent, Natural-Looking Lip Augmentation Using Hyaluronic Acid Fillers

**DOI:** 10.1093/asjof/ojaf131

**Published:** 2025-10-25

**Authors:** Katherine Goldie, Geneviève Ferland-Caron, Vasanop Vachiramon, Bianca Viscomi, Sonja Sattler

## Abstract

The authors of this study present a standardized approach for achieving consistent, natural-looking lip augmentation using cohesive polydensified matrix hyaluronic acid (CPM-HA) fillers. Developed by a panel of 5 global experts, 8 core injection techniques were established to guide procedures, including supporting the oral commissures, enhancing the vermillion border, increasing lateral lip show, increasing vertical height and eversion, improving lip curvature, enhancing lateral structure, and defining the Cupid's bow. The approach outlines a 5-step process: patient interview, documentation, anatomical assessment, lip structure assessment, and treatment, promoting results that are tailored to the patient's unique aesthetic goals and anatomy. Two examples of the application of these core principles are highlighted through the Beautification and Restoration pathways, chosen based on patient-specific needs. Beautification enhances existing lip features, whereas Restoration rebuilds structure lost to aging. Three case studies illustrate how these core principles and techniques are applied in practice, demonstrating how individual patient requirements guide treatment. Case 1 involves a 45-year-old patient seeking subtle lip enhancement. Case 2 outlines the treatment of a 39-year-old patient aimed at restoring lip proportions. Case 3 highlights the treatment for a 57-year-old patient requiring structural support. This methodology provides a flexible, structured framework that adapts to individual patient needs, promoting safe, effective, and consistent outcomes.

**Level of Evidence**: 5 (Therapeutic) 
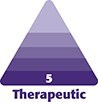

The demand for lip augmentation with soft-tissue fillers continues to grow across various demographics.^1^ However, the variety of available products and techniques may often leave practitioners uncertain about expected outcomes. This can result in inconsistent results and a lack of clarity around why specific techniques are utilized.

Hyaluronic acid (HA)-based injectable fillers are widely utilized to augment or restore areas such as the lips. Modern HA fillers use chemical cross-linking to form cohesive polydensified matrix hydrogels (CPM-HA, Belotero, Anteis S.A., Plan-les-Ouates, Switzerland), which slow HA clearance and maintain results for 6 to 12 months.^2,3^ The CPM-HA range includes fillers with distinct rheological and physiochemical properties that influence product selection and clinical behavior.^4,5^ Key rheological parameters, including elastic modulus (G′), viscosity, and cohesivity affect how a filler interacts with tissue, determining attributes like lift capacity, spreadability, and integration.^5^ For example, CPM-HA 25.5 mg/mL Intense (CPM-HA I) and Lips Shape (CPM-HA S) exhibit a high G′ and cohesivity, making them suitable for deeper structural support and projection, such as at the oral commissures and lateral lip. In contrast, CPM-HA 22.5 mg/mL Balance (CPM-HA B) and Contour (CPM-HA C) have lower viscosity and improved flow characteristics, offering finer control in more superficial planes, such as along the vermillion border (VB) and Cupid's bow.^5^ Understanding these rheologic distinctions allows practitioners to align product choice with intended anatomical depth and treatment outcome, supporting both aesthetic and functional objectives.

This publication introduces the Universal Lip Integrity, Proportion, and Structure (uLIPS) method, a principled, standardized approach to achieving natural-looking lip augmentation using CPM-HA fillers. Rather than prescribing a branded or 1-size-fits-all technique, uLIPS provides a 5-step clinical framework underpinned by 8 core injection techniques, each mapped to specific anatomical targets and aesthetic outcomes. These techniques are applied through 2 treatment pathways, Beautification and Restoration, that address either the enhancement of naturally favorable lip features or the structural correction of age-related volume loss and shape changes. Although the individual injection techniques within uLIPS may be familiar to experienced practitioners, the innovation lies in unifying them into a cohesive, purpose-driven framework. uLIPS decouples injection decision-making from branded protocols, offering a structured methodology that maps each technique to a defined clinical outcome. This principled approach aims to improve reproducibility, clarity of intent, and patient-specific customization in aesthetic lip augmentation.

Three case studies are presented to demonstrate the practical application of this framework. Importantly, the 8 core techniques equip practitioners with foundational methods and clearly defined augmentation outcomes, ensuring both consistency and predictability in clinical practice.

## METHODS

A global panel of 5 physicians with experience in aesthetic medicine convened in August 2023 to establish a standardized framework for consistent, natural-looking lip augmentation using CPM-HA fillers. The panel comprised dermatologists and aesthetic practitioners from Europe, North America, Latin America, and Asia, offering a diverse range of regional and clinical perspectives. This framework was intended to provide clear guidelines for clinicians to ensure predictable and reproducible outcomes (Video 1).

**Figure 1.**
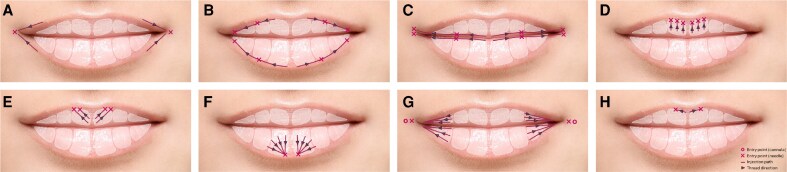
The 8 core injection techniques for lip augmentation. Techniques are selected based on patient anatomy and aesthetic goals to support consistent, natural-looking outcomes using cohesive polydensified matrix hyaluronic acid (CPM-HA) fillers. (A) Corner of the mouth: retrograde threading supports and subtly lifts the oral commissures. (B) Vermillion border architecture: subvermilion threading enhances definition without adding volume. (C) Lip show: Mediolateral threading increases lateral lip exposure. (D) Upper lip height: vertical threads enhance height and eversion. (E) Upper lip curve: oblique threads accentuate natural 3-dimensional curvature. (F) Lower lip height and eversion: fanned threads add volume and eversion. (G) Lateral structure: lateral threading restores symmetry and structure. (H) Cupid's bow: Microdroplet injections refine bow definition.

The panel developed a 5-step framework for patient assessment and treatment, emphasizing the tailoring of lip augmentation procedures to the patient's individual anatomy and aesthetic goals. In addition, 8 core injection techniques were defined to address specific aesthetic concerns, such as commissure support, VB definition, and vertical lip height (Figure 1, Supplemental Figure 1).

### Case Studies

The guidelines were applied to a cohort of 3 patients seeking lip augmentation, with each treatment following the established 5-step framework and 8 core injection techniques. All treatments were performed by Bianca Viscomi, MD, as part of routine clinical care and training. Written informed consent for treatment, photography, and publication of anonymized data and images was obtained from all patients. All patient interactions were carried out according to the principles outlined in the Declaration of Helsinki. Ethical oversight was provided by the responsible physician (B.V.). No formal institutional ethics committee approval was required.

### Recommendations

#### The 5-Step Lip Augmentation Framework

The 5-step framework served as the outcome and methodology of this study, offering a structured approach to tailor treatments to each patient's anatomy and aesthetic goals. This framework was designed to standardize patient assessment, documentation, anatomical evaluation, and lip structure assessment, as well as the application of the 8 core injection techniques. Its application across all patients demonstrated consistent, reproducible results, further validating the method's effectiveness. Case studies illustrate how these guidelines can be applied in clinical practice to achieve tailored, natural-looking outcomes.

The framework begins with a patient interview, where the patient's aesthetic goals are assessed, and psychological concerns, including body dysmorphic disorder or depression, are screened for, because these may impact treatment appropriateness.^6^ Standardized documentation follows, involving static photography from frontal, lateral, and 45° angles in the natural head position and dynamic assessments using video to capture lip movement.^7-9^ The third step involves assessing the anatomical foundations, where skeletal, muscular, and dental structures are evaluated to understand the patient's baseline anatomy, which is crucial for achieving natural-looking results.^9-12^ The assessment of lip structure comes next, focusing on key anatomical landmarks, such as the VB and Cupid's bow, to evaluate lip proportions and symmetry.^13-15^ Finally, based on these assessments, the treatment is carried out using the 8 core injection techniques applied as needed and exemplified by the 2 recommended treatment pathways, Beautification or Restoration.

#### The 8 Core Injection Techniques

The panel defined 8 core injection techniques that provide a structured yet flexible approach to lip augmentation (Figure 1, Supplemental Figure 1). These techniques are selected and combined based on the patient's anatomical needs and aesthetic goals. The “Corner of the Mouth” technique involves retrograde linear threading applied at the oral commissures to lift and support the corners of the mouth, creating a subtle upturn that improves lip symmetry and reduces sagging. “Vermillion border architecture” (VB architecture) technique uses linear threading along the sub-VB to enhance lip definition and structure without adding significant volume, improving the lip contour while maintaining a natural look. To increase lateral lip exposure, the “Lip show” technique involves threading from the medial to lateral sections of the VB, highlighting the natural shape of the lips and enhancing fullness without overprojection.

The “Upper lip height” technique uses retrograde vertical threading to increase vertical lip height and improve eversion, creating a fuller appearance, particularly for patients with flat or thin lips, without altering the natural curvature. For patients seeking to enhance the curvature of the lips, the “Upper lip curve” technique applies oblique retrograde threading to accentuate the 3-dimensional curve of the lips, particularly in the upper lip, contributing to a more defined, fuller shape. The “Lower lip height and eversion” technique fans retrograde threads from a central entry point to simultaneously enhance both lip height and curvature, providing a well-rounded aesthetic.

For patients requiring structural improvements without affecting vertical height, “Lateral structure” technique uses linear retrograde threading in the lateral lip compartments to improve structure and fullness, addressing thinning or asymmetry while maintaining the lips' natural height and curvature. Lastly, the “Cupid's bow” technique uses microdroplet injections along the upper lip's midline to sharpen the definition of the Cupid's bow, enhancing the shape without creating an unnatural or exaggerated appearance.

#### Treatment Pathways: Beautification and Restoration

**Figure 2.**
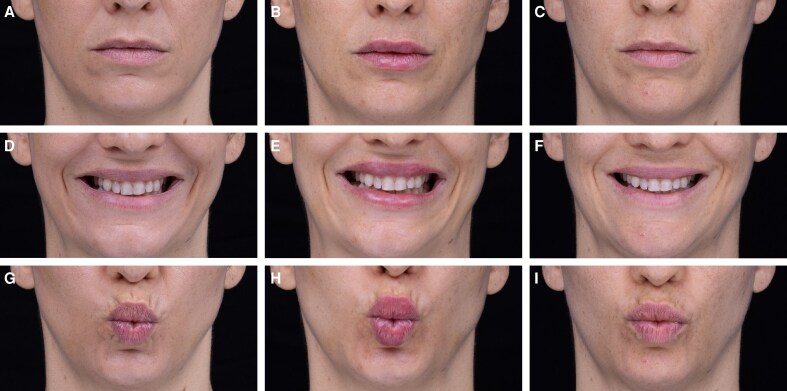
A 45-year-old female patient treated through the Beautification pathway of the Universal Lip Integrity, Proportion, and Structure framework. Images show (A-C) neutral expression; (D-F) smiling; and (G-I) pursed lips with pretreatment images at A, D, G; immediate posttreatment at B, E, H; and 40-day follow-up at C, F, I. Treatment: CPM-HA B/C with 30G needle. Upper lip height: 2 × 0.02 mL threads/side. Upper curve: 2 × 0.025 mL threads/side. Lower lip eversion: 3 × 0.025 mL threads/side. Cupid's bow: 1 × 0.02 mL thread/side (microdroplets). Total volume: 0.37 mL.

**Figure 3.**
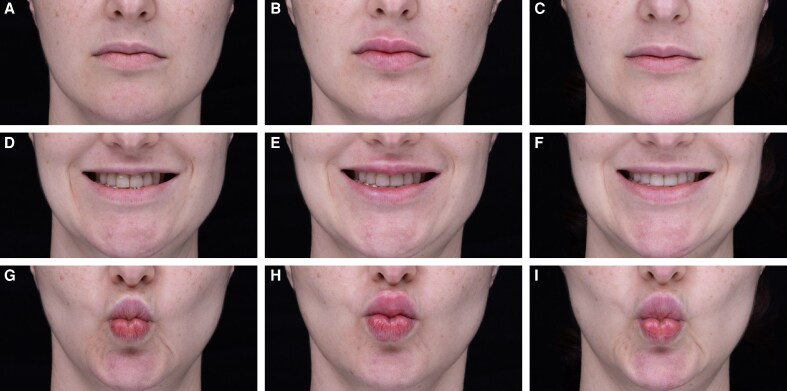
A 39-year-old female patient treated through the Beautification pathway. Images show (A-C) neutral expression; (D-F) smiling; and (G-I) pursed lips, with pretreatment images at A, D, G; immediate posttreatment at B, E, H; and 40-day follow-up at C, F, I. Treatment: CPM-HA B/C with 30G needle. Upper lip height: 3 × 0.02 mL threads/side. Lower lip eversion: 2 × 0.025 mL threads/side. Total volume: 0.22 mL.

Based on the patient's anatomical needs and aesthetic goals, 2 treatment pathways—“Beautification” and “Restoration”—were devised and applied. The Beautification Pathway was selected for patients whose lip structure is already well defined but requires proportional enhancement or improvement in lip features. Key assessment points include the medial tubercle height, upper lip eversion, Cupid's bow definition, and the lower lip's height and eversion. The goal is to maintain existing lip structure, add appropriate volume, and ensure optimal tissue quality without requiring significant structural restoration. This pathway was applied to Cases 1 and 2 (Table 1, Figures 2, 3, Videos 2, 3).

**Table 1.** Beautification Treatment Pathway: Assessment and Injection Summary

Step	1	2	3	4
Lip site	Upper lip height	Upper lip curve	Lower lip height and eversion	Cupid’s bow
Assessment focus	Upper-to-lower lip height ratio Eversion of the upper lip and VB	3D structure Direction of curvature Gingival exposure	Upper-to-lower lip height ratio Eversion of the lower lip	Frontal definition of the Cupid's bow M-shape Lateral definition of the upper lip J-shape
Desired outcome	Increase medial tubercle vertical height and eversion No curvature	Curvature	Increase vertical height and eversion	Cupid bow definition
Technique	Retrograde vertical linear threads	Retrograde oblique threads	Retrograde linear threads Fanning from each entry point	Retrograde linear threads Series of microdroplets (teardrop)
Volume per thread	≤0.025 mL	≤0.025 mL	≤0.020 mL	≤0.020 mL
Total maximum volume	≤0.30 mL	≤0.30 mL	—	—
Entry	1 mm below the VB Multiple entries in the medial tubercle	1 mm below the VB Multiple entries lateral to the Glogau–Klein points within the medial tubercle	1 mm above the VB 2 entry points in lower medial tubercle	Sub-VB entries at Glogau–Klein points
Tool				
CPM-HA I/S	27G needle	27G needle	27G needle	27G needle
CPM-HA B/C	30G needle	30G needle	30G needle	30G needle

All injections should be in the anterior compartment (AC). CPM-HA, cohesive polydensified matrix hyaluronic acid; VB, vermillion border

**Figure 4.**
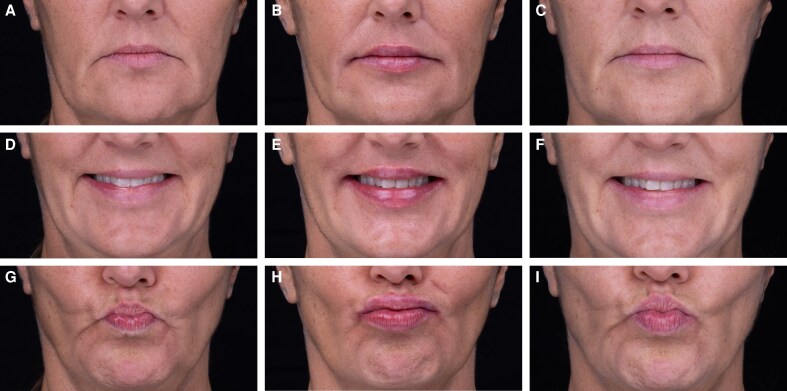
A 57-year-old female patient treated through the Restoration pathway of the Universal Lip Integrity, Proportion, and Structure framework. Images show (A-C) neutral expression; (D-F) smiling; and (G-I) pursed lips with pretreatment images at A, D, G; immediate posttreatment at B, E, H; and 40-day follow-up at C, F, I. Treatment included CPM-HA I/S and B/C using a combination of a 27G needle, a 30G needle, and a 25G cannula. Corner of mouth: 0.08 mL (0.02 mL/quadrant). Vermillion border: 0.24 mL (3 × 0.02 mL/side). Lip show: 0.20 mL (0.05 mL/quarter). Upper lip height: 0.12 mL (3 × 0.02 mL/side). Upper curve: 0.05 mL (1 × 0.025 mL/side). Lower lip eversion: 0.12 mL (3 × 0.02 mL/side). Lateral structure: 0.20 mL (lower lip: 2 × 0.025 mL/side; upper lip: 1 + 3 × 0.025 mL). Total volume: 1.01 mL.

In cases where structural loss is present, particularly in patients over 40 or those with congenital issues, the Restoration pathway is utilized. This pathway addresses areas such as the oral commissures, VB architecture, lip show, and lateral structure before volumization or beautification. The Restoration pathway aims to rebuild lip structure, increase support, and enhance the overall aesthetic appearance. This was applied in Case 3, where structural restoration was necessary before achieving optimal volumization (Table 2, Figure 4, Video 4).

**Table 2.** Restoration Treatment Pathway: Assessment and Injection Summary

Step	1	2	3	4	5	6	7
Lip site	Corner of mouth	VB architecture	Lip show	Upper lip height	Upper lip curve	Lower lip height and eversion	Lateral structure
Assessment focus	Underlying foundations Direction of oral commissures	Underlying foundations VB definition White roll height	Exposure of the lips red vermillion at rest and smiling	Upper-to-lower lip height ratio Eversion of the upper lip and VB	3D structure Direction of curvature Gingival exposure	Upper-to-lower lip height ratio Eversion of the lower lip	Lateral lip architecture Lateral lip height proportional to medial tubercles
Desired outcome	Corner of mouth support Upturn of oral commissures	VB structure	Increase lateral lip show	Increase medial tubercle vertical height and eversion No curvature	Curvature	Increase vertical height and eversion	Lateral structure without height
Treatment							
Technique	Retrograde linear threading Immediately inferior to VB	Retrograde linear threading Lateral to medial following sub-VB line	Retrograde linear threading Fading filler quantity as progressing medial to laterally	Retrograde vertical linear threads	Retrograde oblique threads	Retrograde linear threads Fanning from each entry point	Needle; 2-3 linear retrograde threads in lateral and middle compartments Cannula; Tapering of product, injecting as moving from mid to lateral compartments
Volume per thread	≤0.020 mL	**—**	**—**	≤0.025 mL	≤0.025 mL	≤0.020 mL	≤0.025 mL
Volume maximum	**—**	−0.1-0.2 mL per lip quarter	−0.05-0.1 mL per lip quarter	≤0.30 mL	≤0.30 mL	—	≤0.30 mL
Entry	2 mm lateral to commissure Relaxed open mouth	Multiple entries along sub-VB line	2 mm lateral of commissures In line with wet/dry border	1 mm below the VB Multiple entries in medial tubercle	1 mm below the VB Multiple entries lateral to Glogau–Klein points	1 mm above VB 2 entry points in the lower medial tubercle	2 mm (needle) laterally from commissures 5 mm (cannula) laterally from commissures
Tool							
CPM-HA I/S	27G needle	—	25G cannula	27G needle	27G needle	27G needle	
CPM-HA B/C	—	30G needle OR 25G cannula	—	30G needle	30G needle	30G needle	30G needle OR 25G cannula

All injections should be in the anterior compartment (AC). CPM-HA, cohesive polydensified matrix hyaluronic acid; VB, vermillion border.

#### Injection Techniques and Implementation

For both pathways, the 8 core injection techniques were applied to achieve the desired outcomes in a structured manner. The anterior compartment (AC) was the primary injection plane for all techniques (Video 1).^12^ Injection of CPM-HA fillers into the AC allows for uniform distribution through the reticular dermis, integrating with collagen bundles while remaining well tolerated.^3,16^ The superficial AC plane avoids the orbicularis muscle and labial vessels, reducing risk of intravascular complications, and has demonstrated durable aesthetic results for up to 6 to 12 months.^2,3,17^

Depending on the technique, a needle or cannula was selected, along with the appropriate tool size (eg, a 30G needle for precision, a 27G needle for broader volumization), ensuring optimal depth and product distribution. The product was uniformly injected into the mid-dermal area of the AC, allowing for a natural-looking volume and contour. The needle/cannula should be inserted at a 10° angle with the bevel up. At the superficial AC, the tool should be visible.^18^

Key considerations included ensuring the appropriate tapering of the product along the wet/dry border to avoid overcorrection and carefully massaging the area postinjection to prevent filler extrusion or nodule formation. CPM-HA I/S was particularly useful for structural support, whereas CPM-HA B/C was applied for subtle enhancements, especially along the VB and Cupid's bow.

#### Case 1

A 45-year-old female requested a subtle enhancement without significant volume change. The Beautification pathway was selected, focusing on maintaining the integrity of the vermilion border, supporting the oral commissures, and improving overall lip texture and definition. CPM-HA B/C was injected using a 30G needle. For the “Upper lip height,” 2 threads of 0.02 mL each were injected on each side. For the “Upper lip curve,” 2 threads of 0.025 mL each were injected on each side. For the “Lower lip height and eversion,” 3 threads of 0.025 mL each were injected to each side. For the “Cupid's bow,” a single thread of 0.02 mL was injected into each side in a series of microdroplets. The immediate results showed a subtle yet effective improvement in lip curvature and structure. The patient was satisfied with the natural look, and at the 40-day follow-up, these subtle enhancements persisted, showing notable improvements in skin quality (Figure 2, Video 2).

#### Case 2

A 39-year-old female sought proportional correction with enhanced upper lip curvature. The Beautification pathway was utilized to restore lip height and define the Cupid's bow without increasing lower lip volume. CPM-HA B/C was injected using a 30G needle for each treatment. For “Upper lip height,” 3 threads of 0.02 mL each were injected per side, along the upper lip's VB. On proactive examination, “Upper lip curve” did not require enhancement. Treatment of the “Lower lip height and eversion” was chosen, and 2 threads of 0.025 mL each were injected per side. This treatment aimed to enhance the contour of the upper lip and maintain a balanced proportion between the upper and lower lips. The 40-day follow-up showed sustained results, with the patient's lips appearing natural and balanced (Figure 3, Video 3).

#### Case 3

A 57-year-old female presented with structural loss because of aging, requiring a combination of structural restoration and volumization. The Restoration pathway was selected, prioritizing the rebuilding of the oral commissures, VB, and lateral lip structure. Initially, CPM-HA I/S was injected using a 27G needle for the “Corner of mouth” oral commissure support, with a single thread of 0.02 mL to both the upper and lower lip commissures on each side. For “VB architecture,” CPM-HA B/C was injected using a 30G needle with 3 threads of 0.02 mL along the upper and lower VB on both sides. For “Lip show,” CPM-HA I/S was injected using a 25G cannula with a single thread, injecting a total of 0.05 mL per lip quarter, slowly reducing the amount injected as the cannula moved laterally. For “Upper lip height,” CPM-HA B/C was injected with a 30G needle, with 3 threads of 0.02 mL each, per side. For “Upper lip curve,” CPM-HA B/C was injected with a 30G needle, with a single thread of 0.025 mL each, per side. For “Lower lip height and eversion,” CPM-HA I/S was injected with a 27G needle, with 3 threads of 0.02 mL each, per side. For “Lateral structure,” CPM-HA B/C was injected with a 30G needle, with 2 threads of 0.025 mL each to both sides of the lower lip, and a single or 3 threads to the left and right side of the upper lip, respectively. At the 40-day follow-up, results demonstrated consistent improvement in lip height, volume, and skin quality, with the commissure upturn still visible (Figure 4, Video 4).

### Additional Considerations From the uLIPS Guideline

Healthcare providers should regularly reassess the patient's anatomy, tissue quality, and lip proportions throughout the lip augmentation procedure. Some steps in the Beautification and Restoration pathways may become unnecessary as treatment progresses, allowing providers to skip or adjust steps based on real-time feedback. Continuous communication with the patient is essential to ensure expectations and outcomes align.

Both static and dynamic evaluations of lip movement should be performed during treatment. If an injection step proves redundant after assessment, it can be omitted while maintaining the prescribed order of treatments for optimal results. For more complex procedures, splitting treatments into multiple sessions can help manage swelling and improve outcomes by allowing better product distribution and adjustments.

Vertical injections should taper toward the VB, concentrating volume near the wet/dry border for a natural look. Providers should mold the product immediately after each injection to ensure even distribution and avoid nodule formation. CPM-HA I/S should be held in place for at least 30 s, and a small amount of product should be retained in the syringe for final touchups. In cases where injections are too superficial, corrective techniques, such as postinjection massage, can safely address issues like product maldistribution.^19^

Patients should be informed that postprocedure swelling will subside within 24 to 48 h, with results becoming clearer as the lips settle. Neurotoxins, such as incobotulinumtoxin type A, can be utilized to relax the orbicularis oris muscle, reduce a “gummy smile,” and treat perioral wrinkles, complementing the lip augmentation.^20^ Finally, it is crucial not to overload the injection site. The recommended volume should not exceed 2 mL per session, with the product reserved for minor adjustments as needed.

## DISCUSSION

The 8 core injection techniques outlined in this manuscript provide a foundational and adaptable approach to lip augmentation, emphasizing consistency and safety. By focusing on anatomical principles and tailoring clinical outcomes, these guidelines offer a structured yet flexible method for achieving natural-looking results. The Beautification and Restoration pathways illustrate how these core techniques can be applied based on individual needs, demonstrating that the underlying principles remain adaptable to various cases.

A significant strength of the guidelines is their emphasis on consistency and safety, particularly through the recommendation to inject only into the AC.^3,17^ This approach reduces the risk of complications by avoiding vital vascular structures and ensuring more predictable outcomes. Additionally, by limiting the quantity of filler utilized per injection and maintaining product placement in safe anatomical zones, practitioners can minimize the risk of overcorrection or adverse effects such as nodules or vascular compromise. These principles promote both effective and safe practices, which are particularly important in a delicate area like the lips.

The method also empowers practitioners to make informed, tailored decisions based on a patient's unique anatomy and aesthetic goals. Unlike branded techniques that often prescribe a rigid, step-by-step approach, the uLIPS method encourages dynamic decision making during procedures. Real-time assessments of tissue response and patient-specific variations enable healthcare providers to adjust their technique in response to evolving conditions during treatment, ultimately leading to a more natural-looking result. Furthermore, the structured assessment steps, such as comprehensive photography, static and dynamic evaluations of lip movement, and the standardized assessment of lip structure, ensure that each procedure is well-documented and reproducible. This method allows for tracking progress over time, which is especially valuable for cases requiring multiple treatments or follow-up sessions.

Although this flexibility offers meaningful advantages, it also introduces potential challenges. The uLIPS framework assumes a high level of expertise in facial anatomy and injection techniques, and outcomes may vary in less experienced hands. Moreover, although the technique emphasizes standardized photographic documentation and clinical assessment, the results presented here were evaluated solely by the treating physicians, without independent observers or validated patient-reported outcome measures. As such, the findings should be interpreted as illustrative rather than definitive.

In addition, the methodology was developed by a panel of 5 global experts and is exemplified through 3 case examples. These cases demonstrate practical application but do not constitute a formal clinical study. The limited sample size restricts the ability to draw generalizable conclusions or conduct statistical comparisons. Future research should include larger, controlled studies with long-term follow-up and third-party evaluation to assess the reproducibility and safety of the uLIPS framework in broader clinical practice.

It is also important to recognize that uLIPS does not directly compare its recommendations to other existing lip augmentation techniques or branded approaches. Although this framework was intentionally designed to move beyond proprietary protocols, further comparative analyses would be valuable in validating the specific advantages and limitations of the uLIPS approach. Finally, as the technique and product recommendations are centered on CPM-HA fillers, regional differences in product availability or regulatory approval may affect implementation. Depending on local product access and practitioner experience, adaptation may be required in practice.

Nevertheless, the structured and modular nature of the uLIPS framework positions it as a promising tool for improving consistency, safety, and outcome predictability in aesthetic lip treatments. By aligning anatomical understanding with defined clinical goals, it offers a reproducible approach that can be tailored to diverse patient presentations while maintaining the flexibility necessary for individualized care.

## CONCLUSIONS

The uLIPS framework provides a structured, principle-based approach to lip augmentation that prioritizes anatomy, safety, and natural-looking outcomes. By outlining 8 core injection techniques applied through 2 adaptable pathways, it offers a practical foundation for new injectors and a back-to-basics reminder for experienced practitioners. This approach moves beyond branded protocols, encouraging clinical judgment and reproducibility in aesthetic outcomes across diverse patient presentations.

## Supplemental Material

This article contains supplemental material located online at https://doi.org/10.1093/asjof/ojaf131.

## Supplementary Material

ojaf131_Supplementary_Data
