# Supplementary material for: Universal Lip Integrity, Proportion, and Structure: A Framework for Achieving Consistent, Natural-Looking Lip Augmentation Using Hyaluronic Acid Fillers
Source: Aesthet Surg J Open Forum. 2025 Oct 25;7:ojaf131. doi: 10.1093/asjof/ojaf131 (PMC12836120; doi:10.1093/asjof/ojaf131)
Supplement: ojaf131_Supplementary_Data [file ojaf131_Supplementary_Data.docx]

|  |  |  | **Supplement 1. Eight Core Techniques and Desired Outcomes for Lip Augmentation** | | | | | |  |
| --- | --- | --- | --- | --- | --- | --- | --- | --- | --- |
|  | | | |  | **Area** | **Assessment** | **Injection Technique** | **Outcome** | |
| 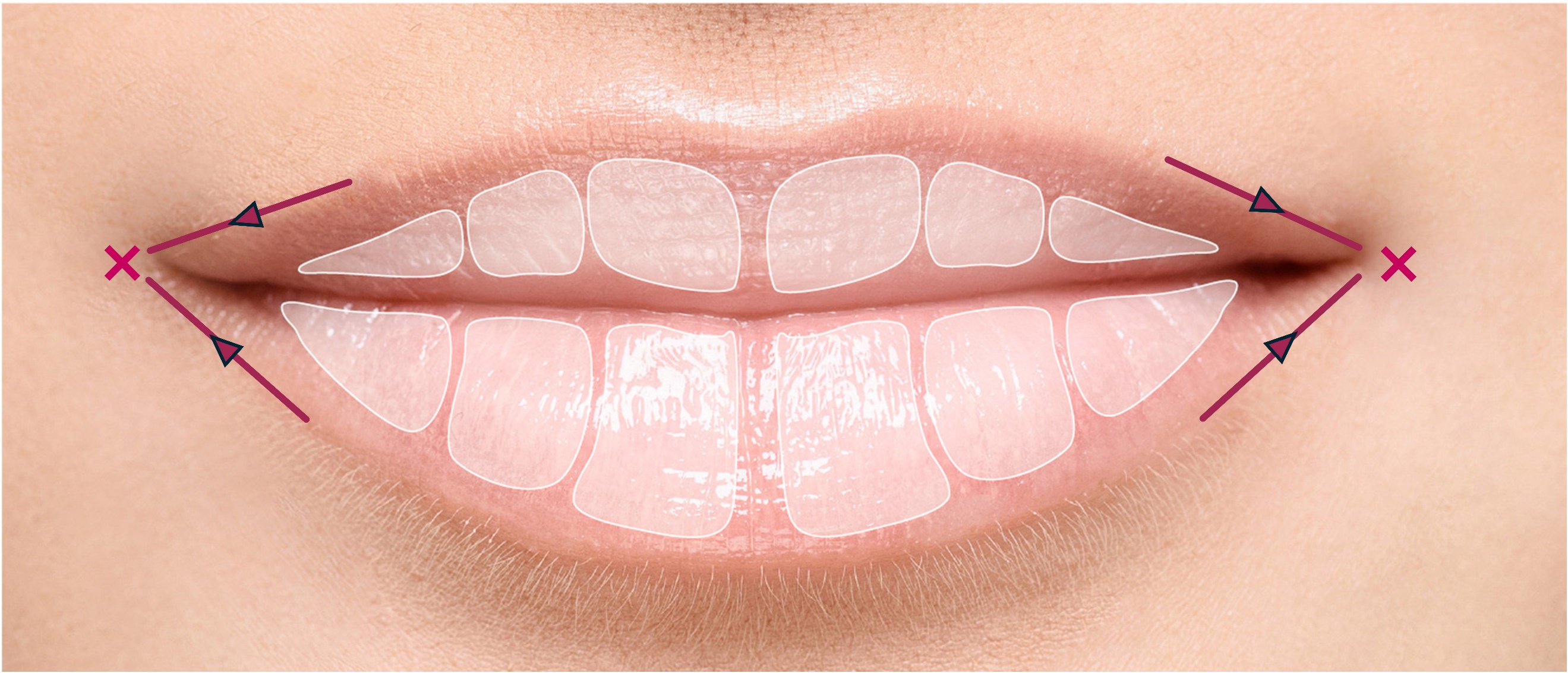 | | | |  | Corner of the Mouth | Assess the severity of oral commissure depression and sagging of facial fat pads. Evaluate the contractility of the depressor anguli oris (DAO) muscle.^14^ | Retrograde linear threading, immediately inferior to the VB | Support the corner of the mouth & upturn of the oral commissures | |
| 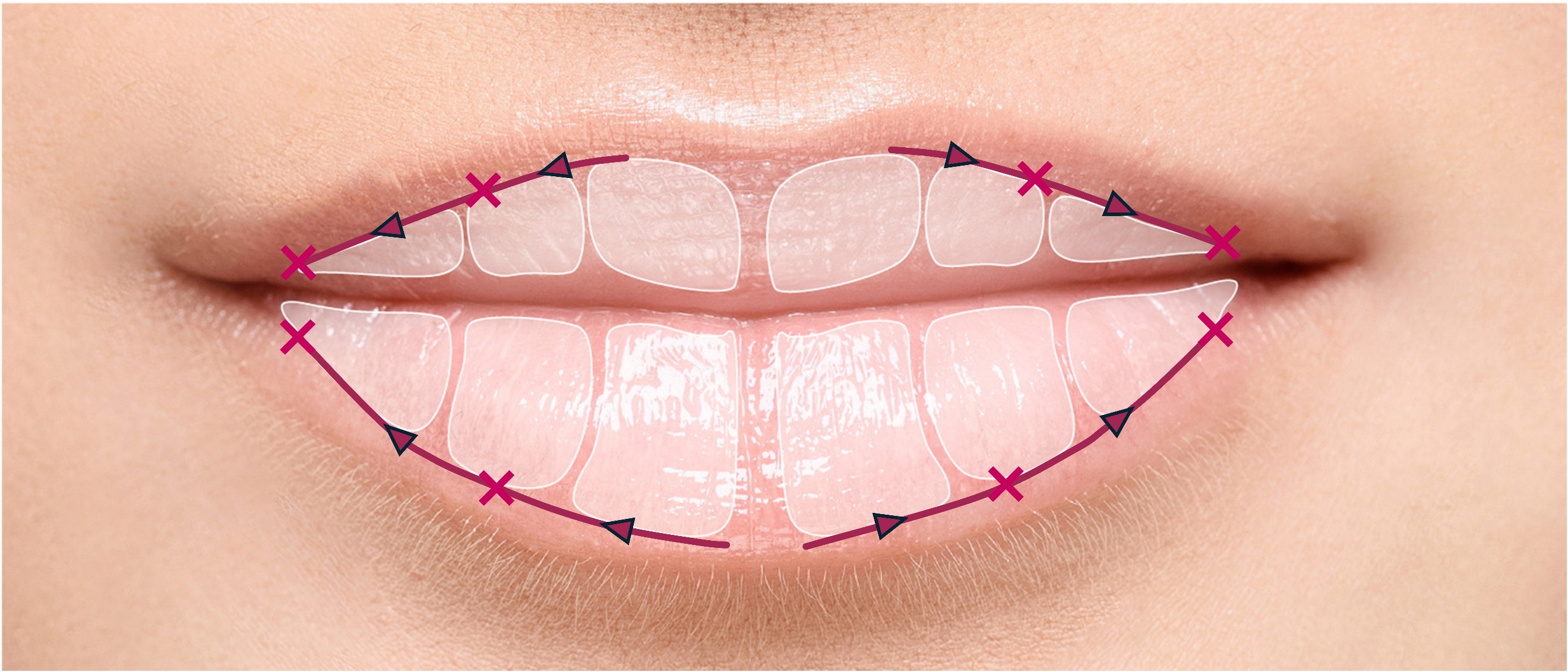 | | | |  | VB Architecture | Evaluate the definition of the VB and signs of fading, determining the need for architectural support without heightening the lip.^14^ | Retrograde linear threading, following the sub-VB line laterally to medially | VB structure | |
| 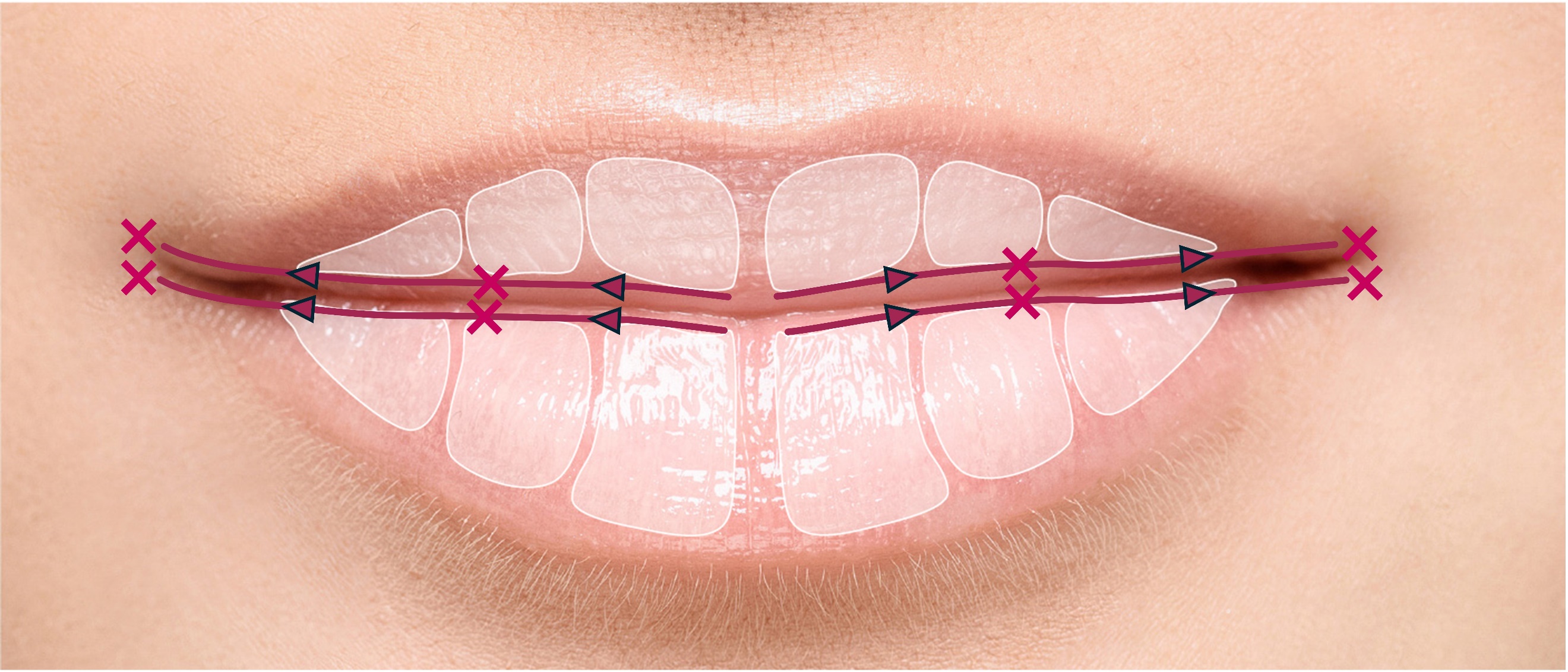 | | | |  | Lip Show | Document the exposure of the red vermillion lip, assessing for enhancement needs. | Retrograde linear threading, progressing medially to laterally | Increased lateral lip show | |
| 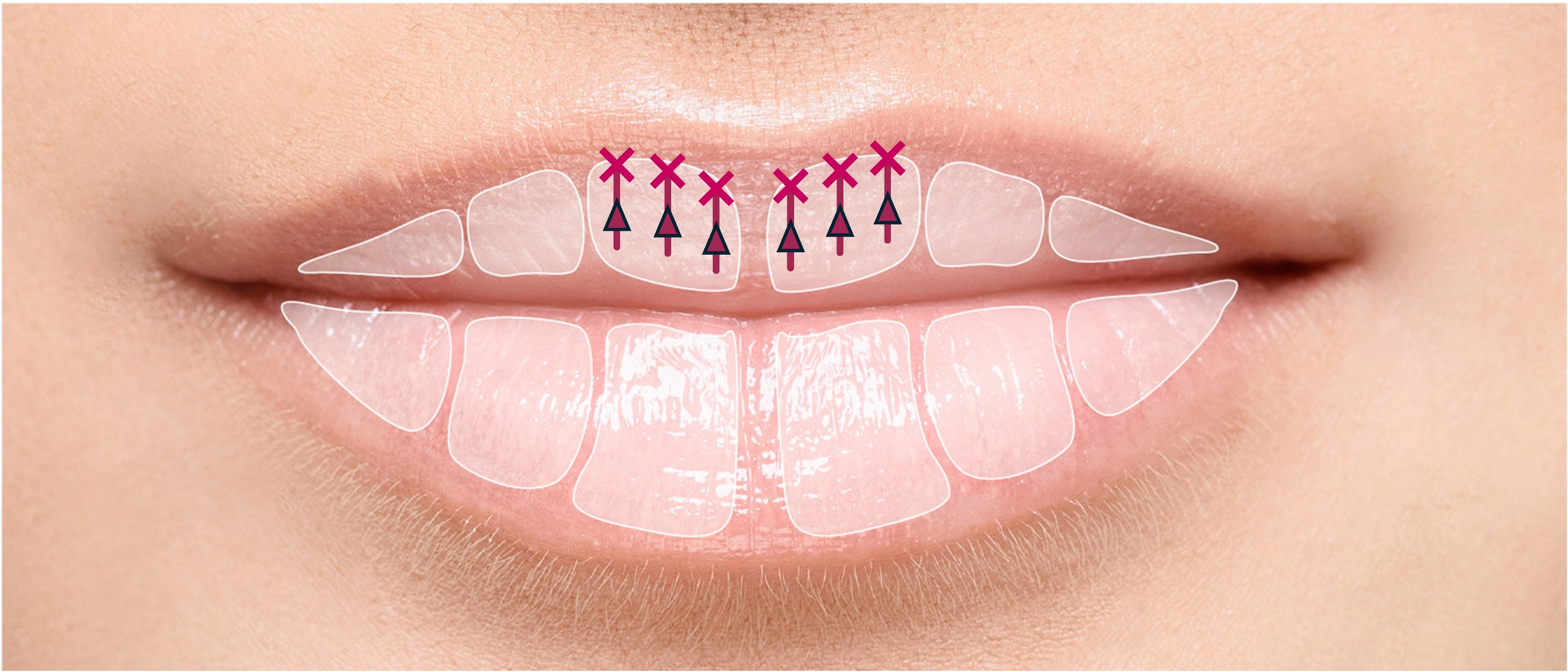 | | | |  | Upper Lip Height | Assess height and eversion, considering the upper to lower lip ratio, with a baseline between 1:1.6 and 1:2.^13^ | Retrograde vertical linear threads | Increase vertical height & eversion without curvature | |
| 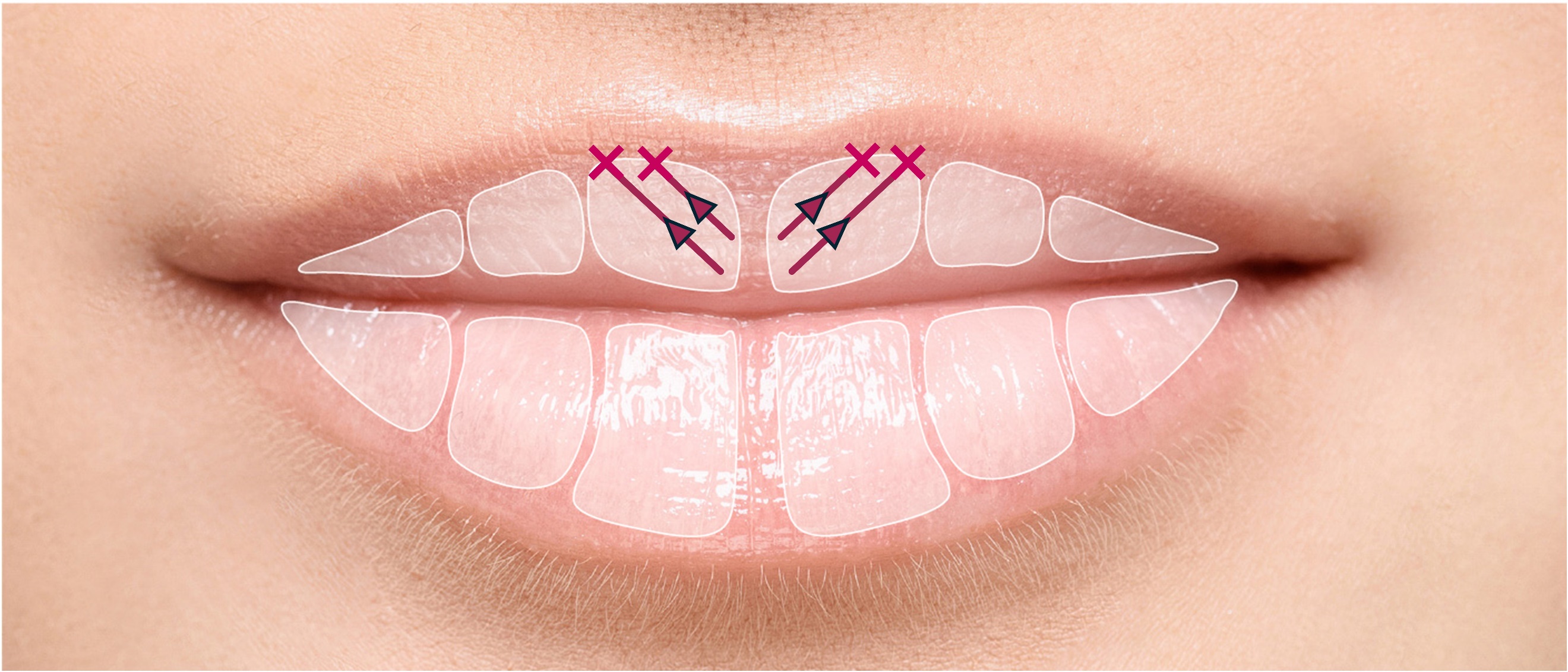 | | | |  | Upper Lip Curve | Examine the three-dimensional curve and directionality, considering maxillary bone and teeth position. | Retrograde oblique threads | Curvature | |
| 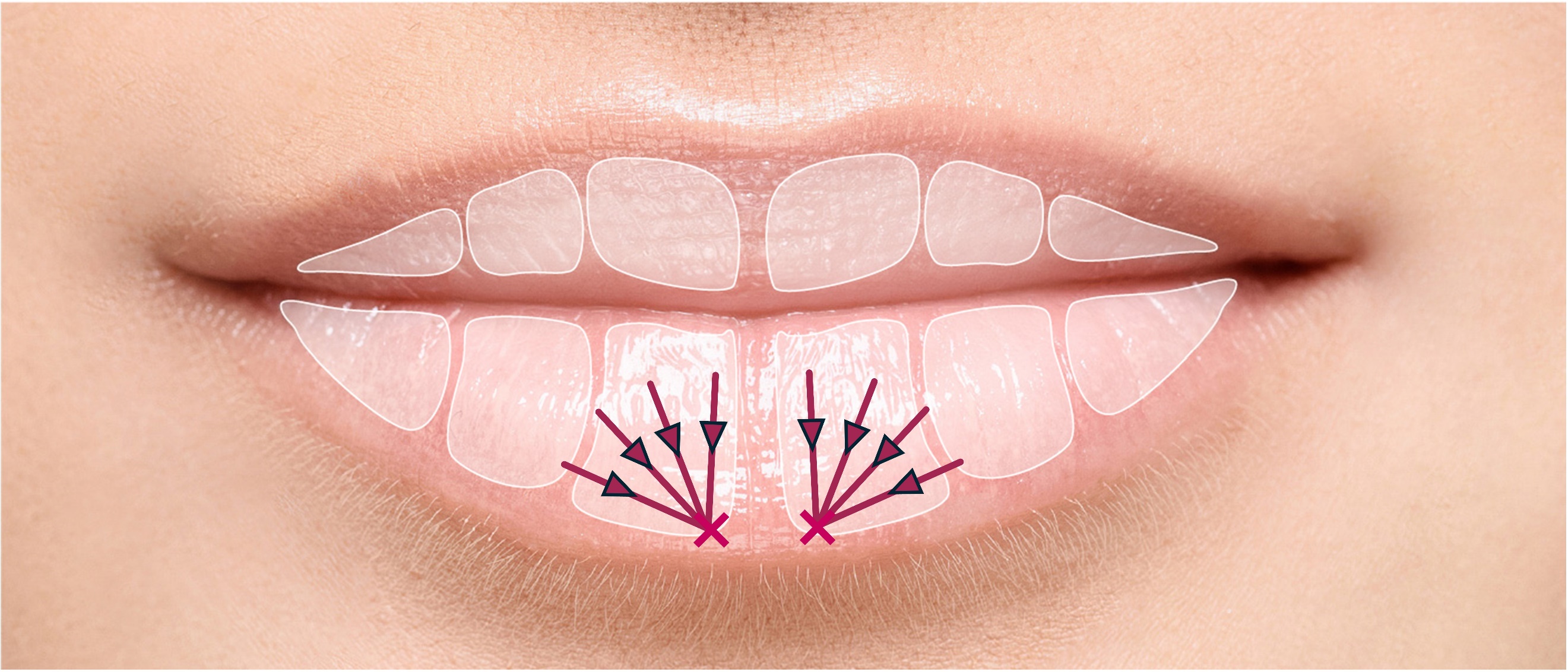 | | | |  | Lower Lip Height & Eversion | Assess height, volume, and eversion. | Retrograde linear threads, fanning from each entry point | Increase vertical height & eversion | |
| 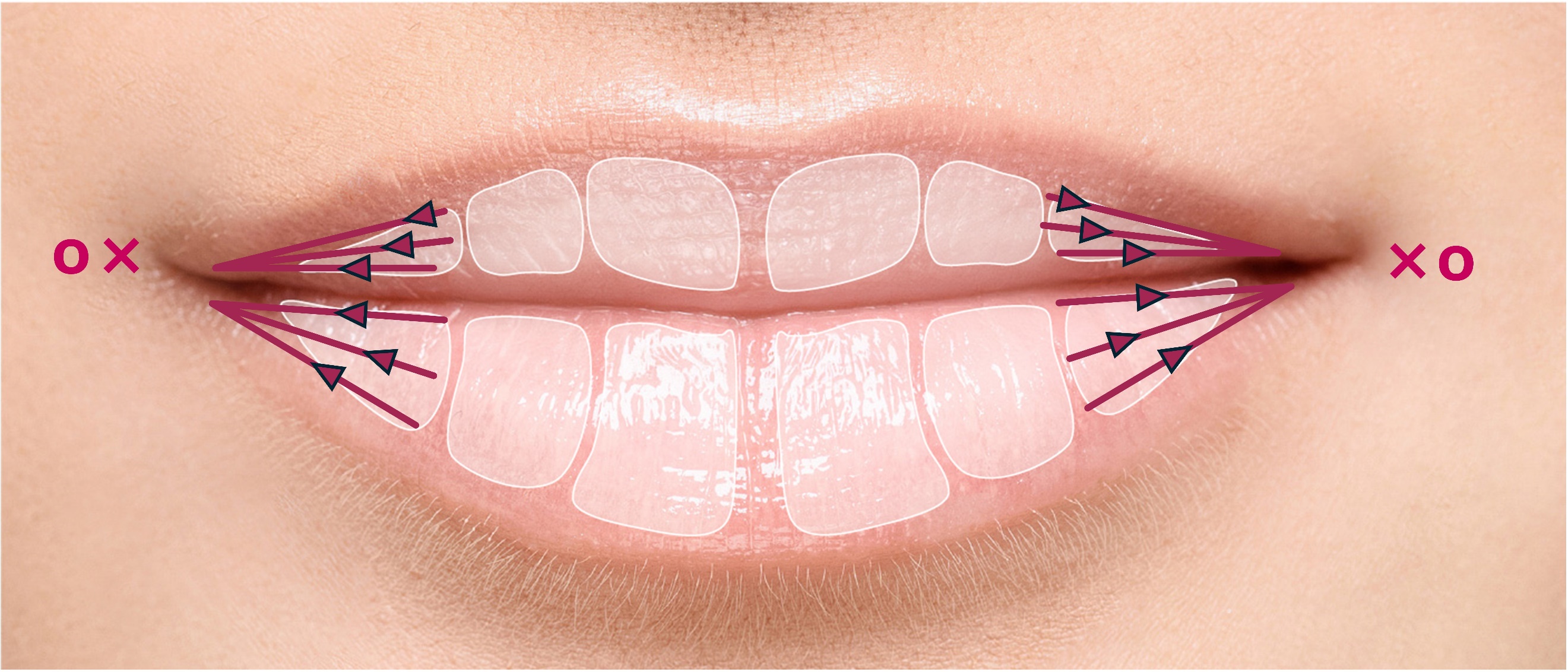 | | | |  | Lateral Structure | Assess for thinning and inward roll, evaluating loss of height and natural curve. | Linear retrograde threads in the lateral and middle compartments | Improve lateral structure without affecting height | |
| 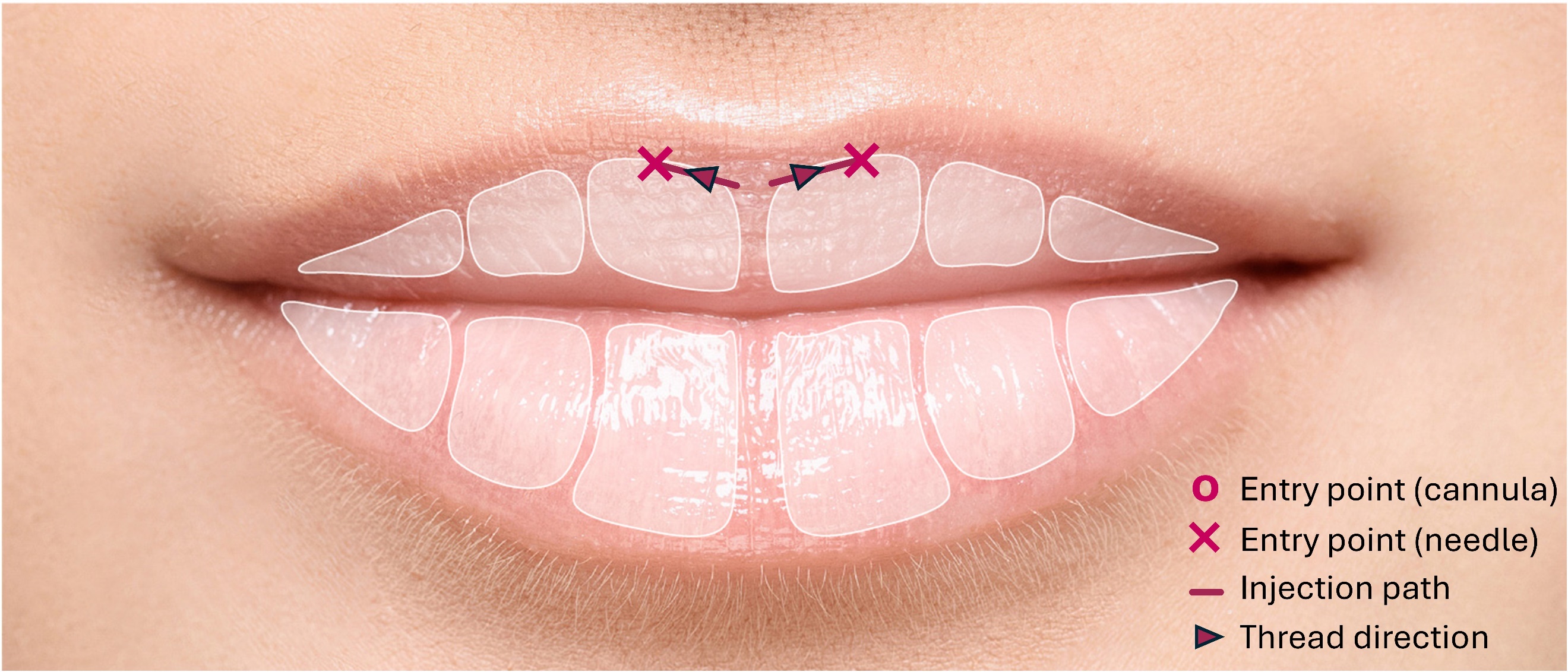 | | | |  | Cupid’s Bow | Measure the prominence and definition of the Cupid’s bow, considering the lip contour from frontal and lateral views.^14^ | Retrograde linear threads with a series of microdroplets | Cupid’s bow definition | |
| VB, vermillion border. X, needle entry point. O, cannula entry point. Line (-), injection path. Arrow (►), thread direction (ante/retro-grade) | | | | | | | | | |
